# Supplementary material for: Unified classification and risk-stratification in Acute Myeloid Leukemia
Source: Nat Commun. 2022 Aug 8;13:4622. doi: 10.1038/s41467-022-32103-8 (PMC9360033; doi:10.1038/s41467-022-32103-8)
Supplement: Supplementary file 3 — Description of Additional Supplementary Files [file 41467_2022_32103_MOESM3_ESM.pdf]

Description of Additional Supplementary Files

File Name: Supplementary Data 1
Description: Clinical data in the NCRI AML Cohort (n=2,113).

File Name: Supplementary Data 2
Description: Study targeted panel sequencing bed file, containing information on the genomic co-ordinates of genes, exons and loci (where snp probes were tiled) captured in the panel.

File Name: Supplementary Data 3
Description: List of oncogenic and putative oncogenic mutations in NCRI AML dataset.

File Name: Supplementary Data 4
Description: List of Cytogenetic variables for NCRI AML cohort.

File Name: Supplementary Data 5
Description: Modeling outputs for multivariate Cox transitions analysis for training and validation cohorts. Pvalues are computed with Wald test.

File Name: Supplementary Data 6
Description: Software and packages used for the regression modeling.

File Name: Supplementary Data 7
Description: Different datasets and files used in this analysis

File Name: Supplementary Data 8
Description: Notebook codes used to generate Main Figures, Supplementary Figures
