# Supplementary figures and images for: Unified classification and risk-stratification in Acute Myeloid Leukemia
Source: Nat Commun. 2022 Aug 8;13:4622. doi: 10.1038/s41467-022-32103-8 (PMC9360033; doi:10.1038/s41467-022-32103-8)

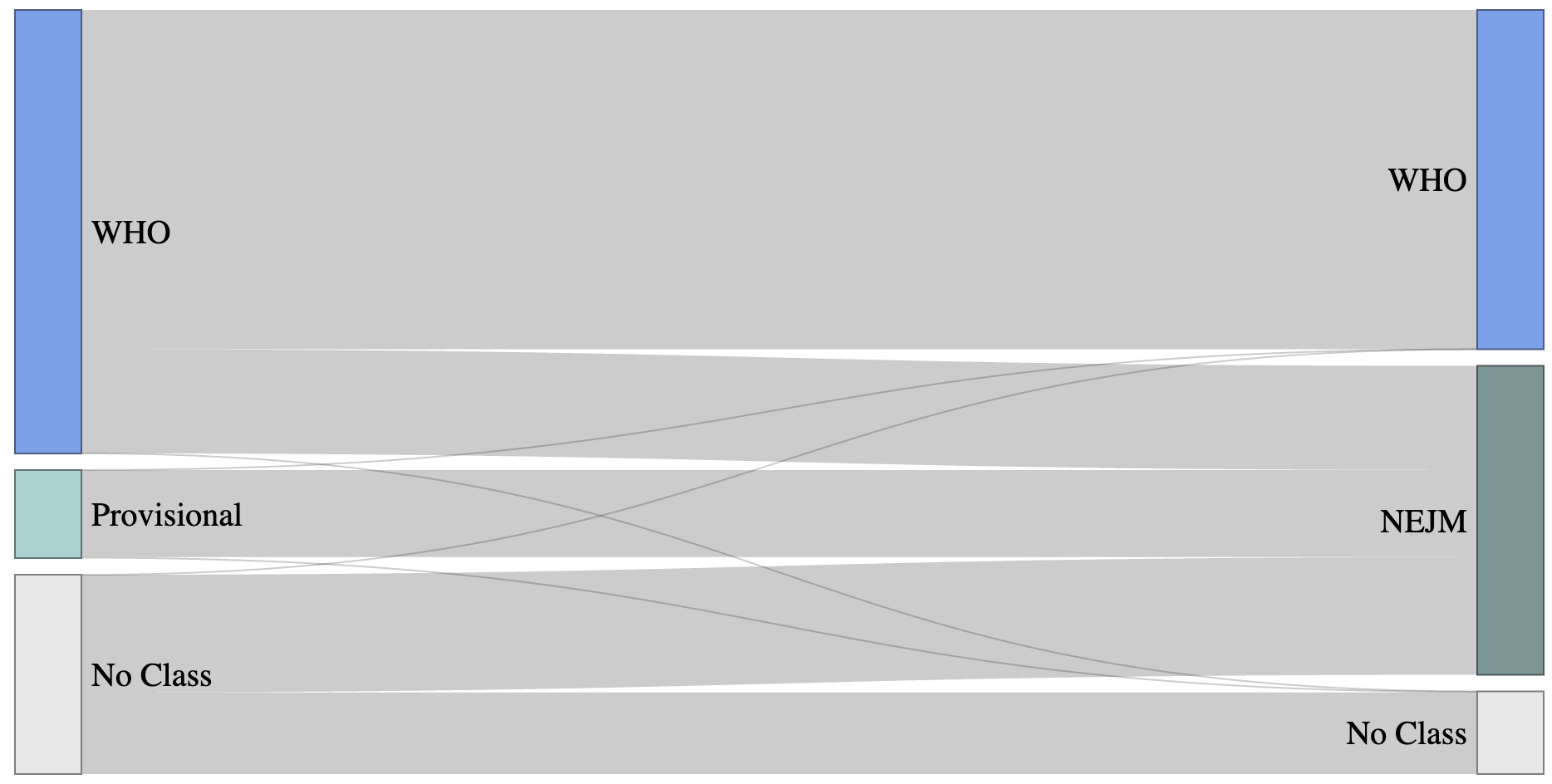

Supplement: Supplementary file 10 — Supplementary Data 7 [file 41467_2022_32103_MOESM10_ESM.zip › data/WHO_NEJM_Sankey.png]

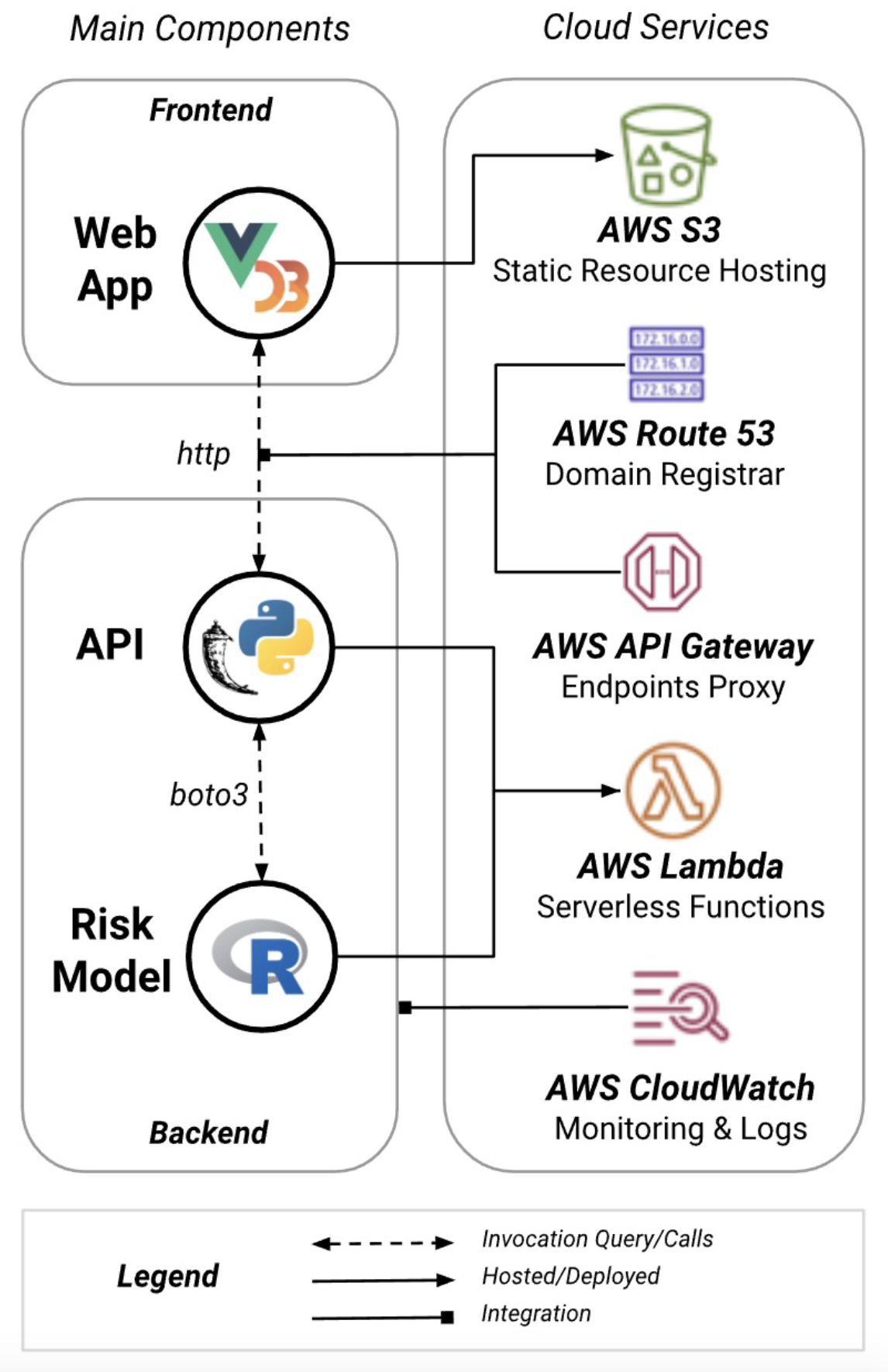

Supplement: Supplementary file 10 — Supplementary Data 7 [file 41467_2022_32103_MOESM10_ESM.zip › data/webapp_infrastructure.png]

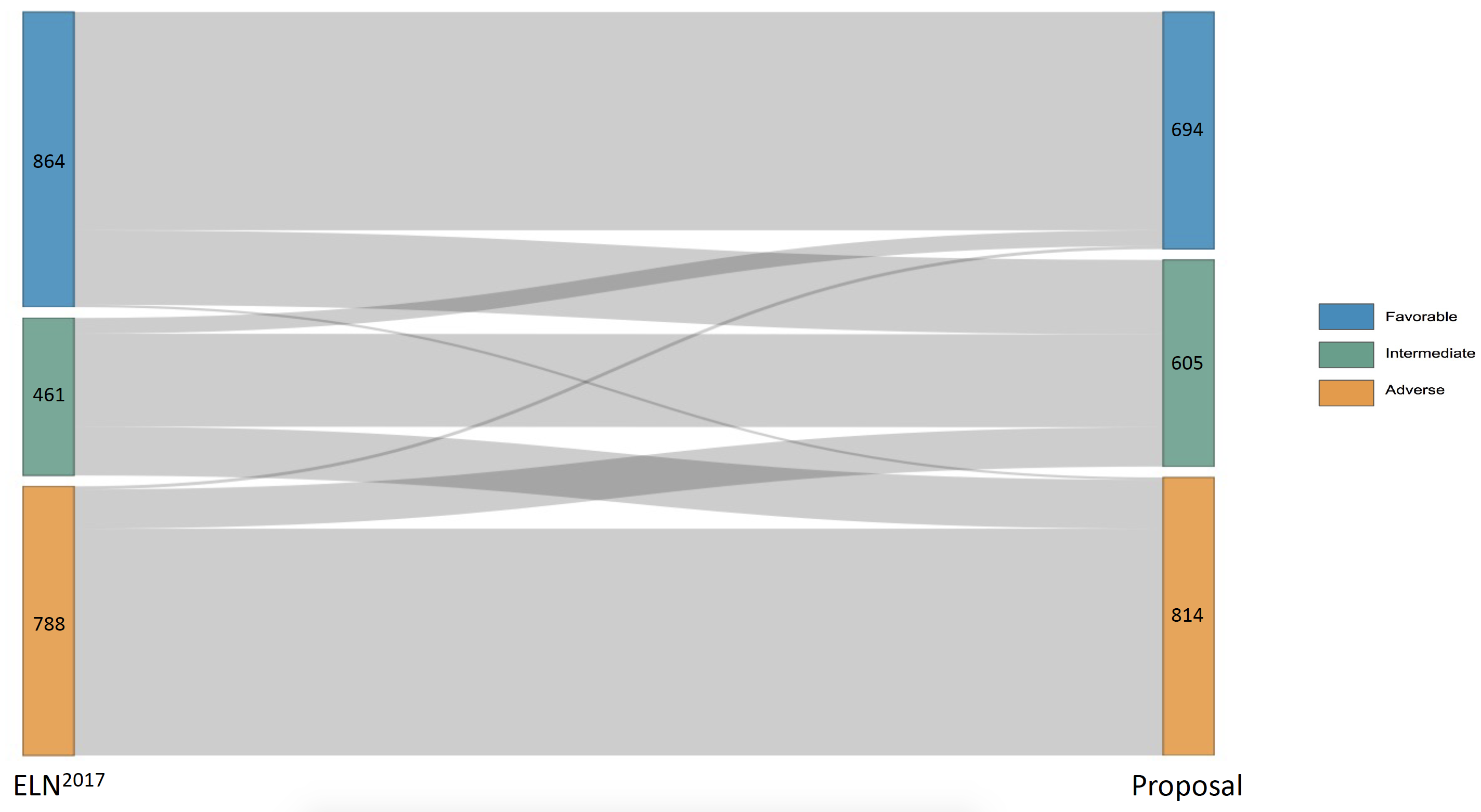

Supplement: Supplementary file 10 — Supplementary Data 7 [file 41467_2022_32103_MOESM10_ESM.zip › data/sankey_plot.png]

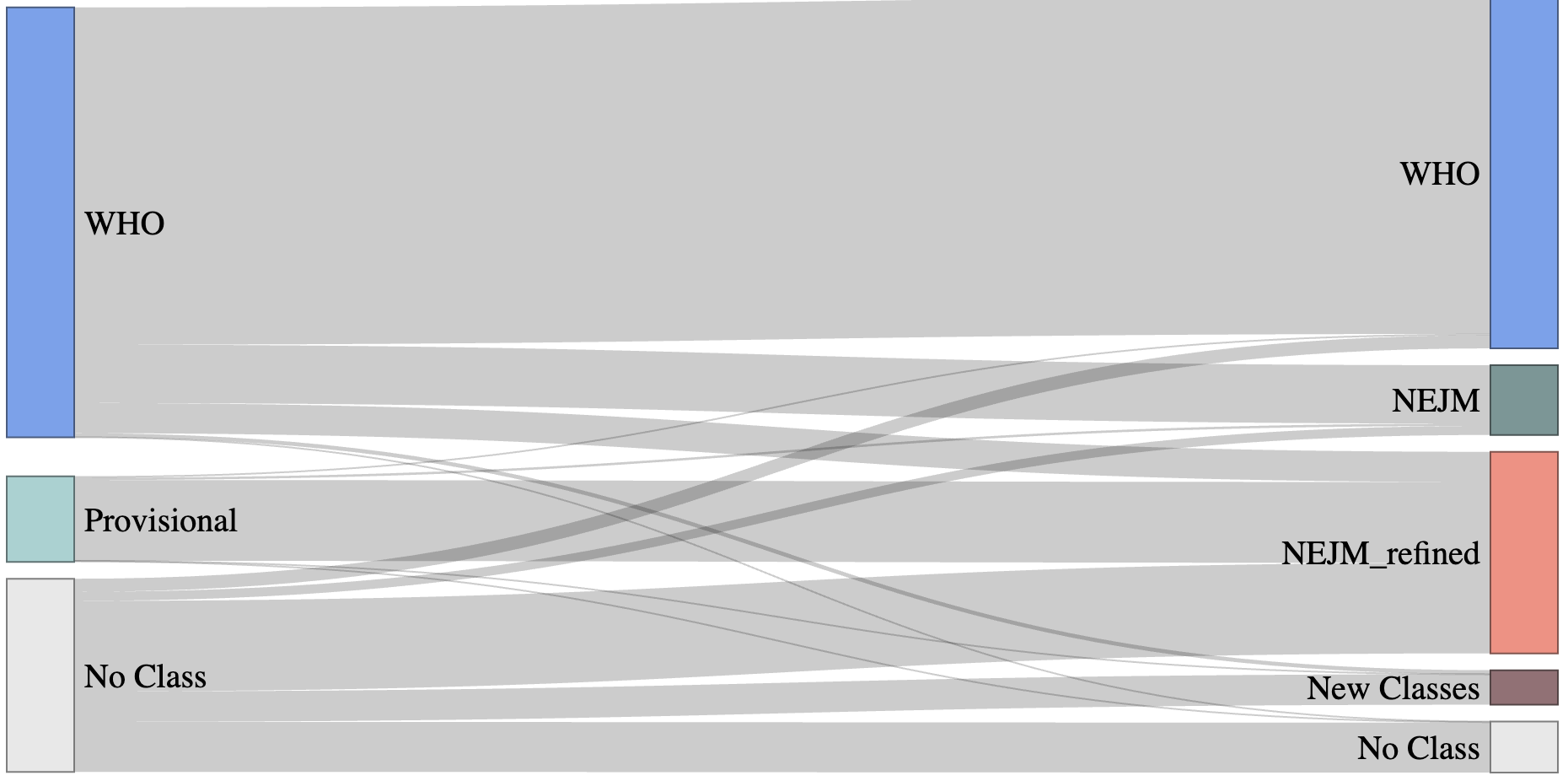

Supplement: Supplementary file 10 — Supplementary Data 7 [file 41467_2022_32103_MOESM10_ESM.zip › data/WHO_MRC_Sankey.png]

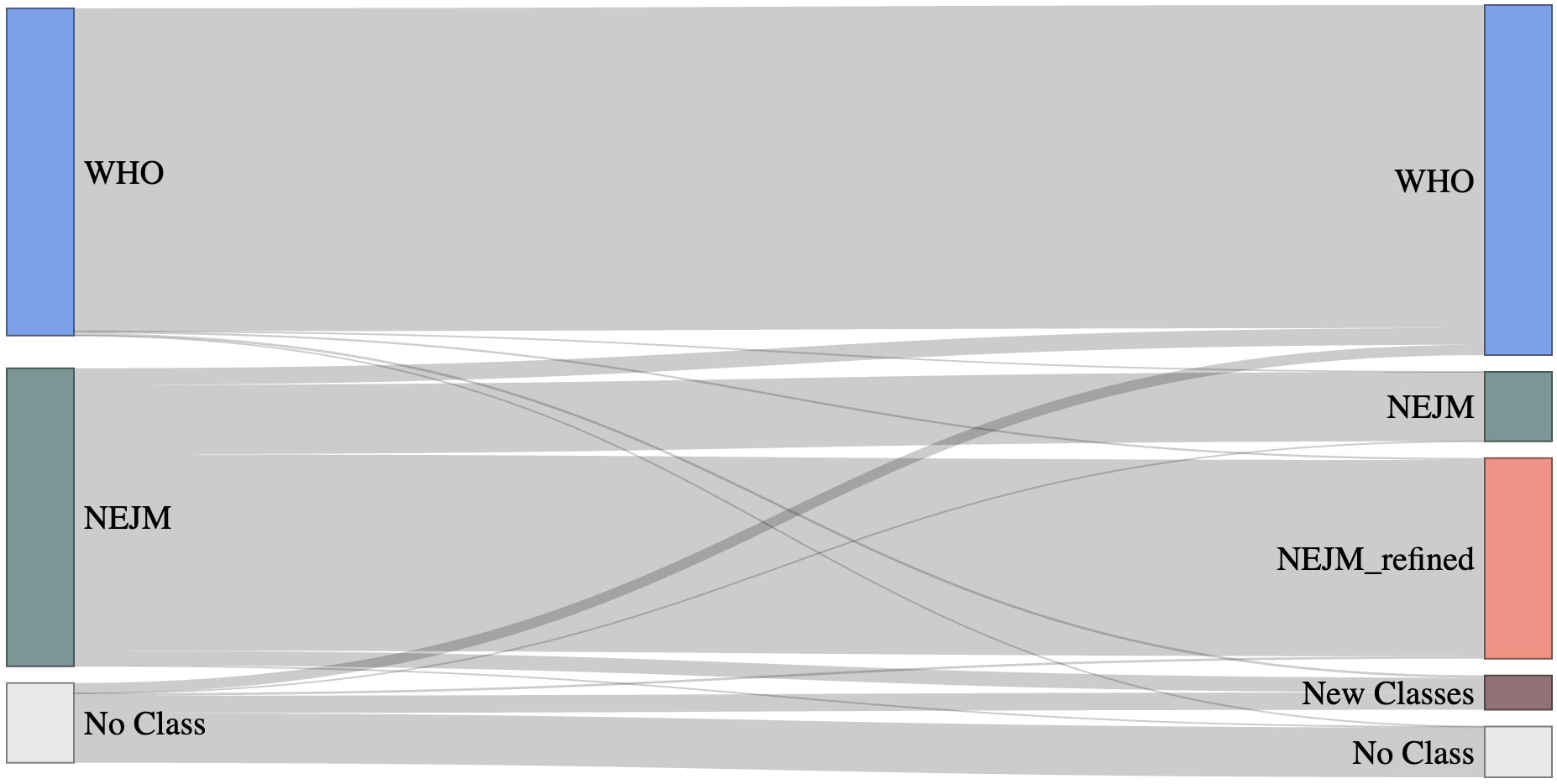

Supplement: Supplementary file 10 — Supplementary Data 7 [file 41467_2022_32103_MOESM10_ESM.zip › data/NEJM_MRC_Sankey.png]

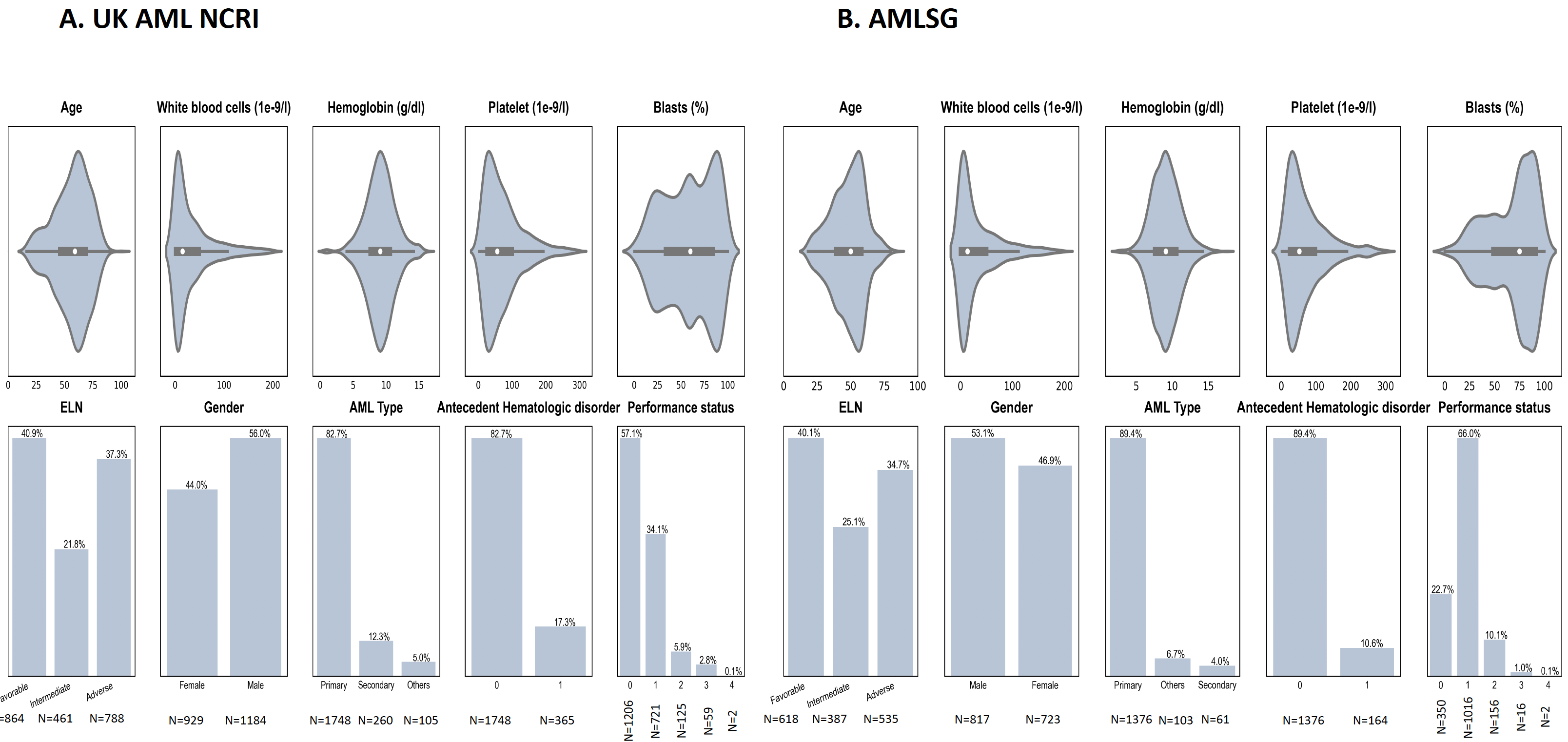

Supplement: Supplementary file 10 — Supplementary Data 7 [file 41467_2022_32103_MOESM10_ESM.zip › data/study_cohort.png]
