# Supplementary material for: Unified classification and risk-stratification in Acute Myeloid Leukemia
Source: Nat Commun. 2022 Aug 8;13:4622. doi: 10.1038/s41467-022-32103-8 (PMC9360033; doi:10.1038/s41467-022-32103-8)
Supplement: Supplementary file 12 — Reporting Summary [file 41467_2022_32103_MOESM12_ESM.pdf]

Corresponding author(s): Elli Papaemmanuil

Last updated by author(s): May 25, 2022

Reporting Summary

Nature Portfolio wishes to improve the reproducibility of the work that we publish. This form provides structure for consistency and transparency in reporting. For further information on Nature Portfolio policies, see our Editorial Policies and the Editorial Policy Checklist.

Statistics

For all statistical analyses, confirm that the following items are present in the figure legend, table legend, main text, or Methods section.

n/a	Confirmed	
☐	☒	The exact sample size (*n*) for each experimental group/condition, given as a discrete number and unit of measurement
☒	☐	A statement on whether measurements were taken from distinct samples or whether the same sample was measured repeatedly
☐	☒	The statistical test(s) used AND whether they are one- or two-sided<br>*Only common tests should be described solely by name; describe more complex techniques in the Methods section.*
☐	☒	A description of all covariates tested
☐	☒	A description of any assumptions or corrections, such as tests of normality and adjustment for multiple comparisons
☐	☒	A full description of the statistical parameters including central tendency (e.g. means) or other basic estimates (e.g. regression coefficient) AND variation (e.g. standard deviation) or associated estimates of uncertainty (e.g. confidence intervals)
☐	☒	For null hypothesis testing, the test statistic (e.g. *F*, *t*, *r*) with confidence intervals, effect sizes, degrees of freedom and *P* value noted<br>*Give P values as exact values whenever suitable.*
☐	☒	For Bayesian analysis, information on the choice of priors and Markov chain Monte Carlo settings
☒	☐	For hierarchical and complex designs, identification of the appropriate level for tests and full reporting of outcomes
☒	☐	Estimates of effect sizes (e.g. Cohen's *d*, Pearson's *r*), indicating how they were calculated

Our web collection on statistics for biologists contains articles on many of the points above.

Software and code

Policy information about availability of computer code

Data collection	No software was used for data collection
Data analysis	Alignment :<br>Raw sequence data were aligned to the human genome (NCBI build 37) using BWA1. Unmapped reads, PCR duplicates and reads mapping to regions outside of the target region (merged exonic regions + 10bp either side of each exon) were excluded from analysis. Bedtools® coverage v2.15.05 was subsequently used to determine the coverage depth at each base. Genes with median target coverage < 20x were removed from the study and samples with median overall coverage < 50x were also excluded from downstream analysis and are not reported in this study.<br><br>Variant calling :<br>Single base, somatic substitutions were called independently in each sample using an in-house algorithm CaVEMan: Cancer Variants through Expectation Maximisation. The algorithm compares sequence data from each tumor sample to an unrelated normal sample and calculates a mutation probability at each base-pair position locus. A number of post-processing filters were applied to improve specificity.<br><br>Quality control (QC) and variant annotation:<br>QC of fastq and bam data were performed with fastQC.<br>From the list of high confident somatic variants, putative oncogenic variants were distinguished from variants of unknown significance (VUS) based on:<br>Recurrence in the Catalogue Of Somatic Mutations in Cancer (COSMIC), in myeloid disease samples registered in cBioPortal or in the study dataset.<br>The inferred consequence of a mutation; where nonsense mutations, splice site mutations and frameshift indels were considered oncogenic for likely tumor suppressor genes (from COSMIC Cancer Census Genes or OncoKB Cancer Gene List).<br>Presence in pan-cancer hotspot databases

Annotation in the human variation database ClinVar
Annotation in the precision oncology knowledge database OncoKB.
Recurrence with somatic presentation in a set of in-house data derived from >6,000 myeloid neoplasms

Statistics:
All statistical analyses were conducted using the R statistical platform (R Core Team 2019) (https://www.r-project.org/) version 3.6.1. Kaplan-Meier estimates were computed using the "survival" R package, relapse risk were estimated using the "cmprsk" R package and Cox proportional hazards regressions were performed using the "coxph" R package and "glmnet" for Cox penalization models. Nonparametric estimated curves of the hazard rate were performed with the "bshazard" R package. Multistate model transitions analysis was performed using "mstate" R package.

For manuscripts utilizing custom algorithms or software that are central to the research but not yet described in published literature, software must be made available to editors and reviewers. We strongly encourage code deposition in a community repository (e.g. GitHub). See the Nature Portfolio guidelines for submitting code & software for further information.

Data

Policy information about availability of data

All manuscripts must include a data availability statement. This statement should provide the following information, where applicable:

- Accession codes, unique identifiers, or web links for publicly available datasets
- A description of any restrictions on data availability
- For clinical datasets or third party data, please ensure that the statement adheres to our policy

All raw data has been deposited in the European Genome-Phenome Archive EGAS00001000570
All data will be available with publication at https://github.com/yanistazi/AML_Repo.
Databases used in the study are gnomAD https://gnomad.broadinstitute.org, COSMIC https://cancer.sanger.ac.uk/cosmic, cBioPortal for Cancer Genomics https://www.cbioportal.org, OncoKB Precision Oncology Knowledge Base https://www.oncokb.org, ClinVar https://www.ncbi.nlm.nih.gov/clinvar

Field-specific reporting

Please select the one below that is the best fit for your research. If you are not sure, read the appropriate sections before making your selection.

☒ Life sciences ☐ Behavioural & social sciences ☐ Ecological, evolutionary & environmental sciences

For a reference copy of the document with all sections, see nature.com/documents/nr-reporting-summary-flat.pdf

Life sciences study design

All studies must disclose on these points even when the disclosure is negative.

Sample size	No statistical methods were used to predetermine sample size; all 3,653 (2,113+1,540) available samples that passed quality control were utilized
Data exclusions	We initially considered 2,150 AML patient samples in training, and we excluded 37 patients for missing survival/genomic data and discrepancies.
Replication	We validated results on an independent cohort of 1,540 older AML patients from the UK AML SG cohort (NEJM PMID: 27276561).
Randomization	This is not relevant to the study, no experimental group allocation.
Blinding	Blinding was not relevant to the study, as there was no control and treatment arms involved.

Reporting for specific materials, systems and methods

We require information from authors about some types of materials, experimental systems and methods used in many studies. Here, indicate whether each material, system or method listed is relevant to your study. If you are not sure if a list item applies to your research, read the appropriate section before selecting a response.

Materials & experimental systems

n/a | Involved in the study

- [x] n/a [ ] Antibodies
- [x] n/a [ ] Eukaryotic cell lines
- [x] n/a [ ] Palaeontology and archaeology
- [x] n/a [ ] Animals and other organisms
- [ ] n/a [x] Human research participants
- [ ] n/a [x] Clinical data
- [x] n/a [ ] Dual use research of concern

Methods

n/a | Involved in the study

- [x] n/a [ ] ChIP-seq
- [x] n/a [ ] Flow cytometry
- [x] n/a [ ] MRI-based neuroimaging

Human research participants

Policy information about studies involving human research participants

opulation characteristics	Covariate population characteristics:<br>- Age at diagnosis<br>- Gender<br>- Bone marrow blasts count<br>- Hemoglobin level<br>- Platelet level<br>- Type of AML<br>- Performance status<br>- Treatment (Intensive or not)<br>- Transplant type (subset of cohort)<br>- MRD (subset of cohort)<br>- Complete remission<br>- Relapse<br>- Overall survival
Recruitment	All patients with a diagnosis of acute myeloid leukemia at any of the partner institutions were eligible for and consented for the study. No exclusionary criteria existed. Patient's samples had to be either diagnostic or ascertained prior to the patient receiving disease modifying treatments that could alter the molecular and clonal inferences.
thics oversight	Sample collection was approved by the Wales research ethic commitee protocol number 08/MRE09/29<br>Analysis of the data in this study was approved by MSKCC Institutional Review Board protocol number x20-064

Note that full information on the approval of the study protocol must also be provided in the manuscript.

Clinical data

Policy information about clinical studies

All manuscripts should comply with the ICMJE guidelines for publication of clinical research and a completed CONSORT checklist must be included with all submissions.

Clinical trial registration	Not a clinical trial
Study protocol	*Note where the full trial protocol can be accessed OR if not available, explain why.*
Data collection	*Describe the settings and locales of data collection, noting the time periods of recruitment and data collection.*
Outcomes	*Describe how you pre-defined primary and secondary outcome measures and how you assessed these measures.*
